# Development of a Biosensor to Detect Venom of Malayan Krait (*Bungarus candidus*)

**DOI:** 10.3390/toxins16010056

**Published:** 2024-01-19

**Authors:** Kiattawee Choowongkomon, Janeyuth Chaisakul, Supaphorn Seetaha, Taksa Vasaruchapong, Wayne C. Hodgson, Natchaya Rasri, Katechawin Chaeksin, Sattawat Boonchaleaw, Nattapon Sookprasert

**Affiliations:** 1 Department of Biochemistry, Faculty of Science, Kasetsart University, 50 Ngam Wong Wan Road, Chatuchak, Bangkok 10900, Thailand; kiattawee.c@ku.th (K.C.); supaporn.se@ku.th (S.S.); natchaya.rasr@ku.th (N.R.); 2 Genetic Engineering Interdisciplinary Program, Graduate School, Kasetsart University, Bangkok 10900, Thailand; katechanok.c@ku.th (K.C.); sattawat.bo@ku.th (S.B.); 3 Department of Pharmacology, Phramongkutklao College of Medicine, Ratchawithi Road, Ratchathewi, Bangkok 10400, Thailand; 4 Snake Farm, Queen Saovabha Memorial Institute, Thai Red Cross Society, Bangkok 10330, Thailand; taksa.v@gmail.com; 5 Monash Venom Group, Department of Pharmacology, Biomedical Discovery Institute, Monash University, Clayton, VIC 3800, Australia; wayne.hodgson@monash.edu; 6 Department of Preclinical Science, Faculty of Medicine, Thammasat University, Rangsit Campus, Pathumthani 12120, Thailand

**Keywords:** biosensors, venom, snake, antivenom, detection kit, Malayan krait

## Abstract

Malayan krait (*Bungarus candidus*) envenoming is a cause of significant morbidity and mortality in many Southeast Asian countries. If intubation and specific antivenom administration are delayed, the most significant life-threatening outcome may be the inhibition of neuromuscular transmission and subsequent respiratory failure. It is recommended that krait-envenomed victims without indications of neurotoxicity, e.g., skeletal muscle weakness or ptosis, immediately receive 10 vials of antivenom. However, the administration of excess antivenom may lead to hypersensitivity or serum sickness. Therefore, monitoring venom concentrations in patients could be used as an indicator for snake antivenom treatment. In this study, we aimed to develop a screen-printed gold electrode (SPGE) biosensor to detect *B. candidus* venom in experimentally envenomed rats. The gold electrodes were coated with monovalent Malayan krait IgG antivenom and used as venom detection biosensors. Electrochemical impedance spectrometry (EIS) and square wave voltammetry (SWV) measurements were performed to detect the electrical characterization between *B. candidus* venom and monovalent IgG antivenom in the biosensor. The EIS measurements showed increases in charge transfer resistance (R_ct_) following IgG immobilization and incubation with *B. candidus* venom solution (0.1–0.4 mg/mL); thus, the antibody was immobilized on the electrode surface and venom was successfully detected. The lowest current signal was detected by SWV measurement in rat plasma collected 30 min following *B. candidus* experimental envenoming, indicating the highest level of venom concentration in blood circulation (4.3 ± 0.7 µg/mL). The present study demonstrates the ability of the SPGE biosensor to detect *B. candidus* venom in plasma from experimentally envenomed rats. The technology obtained in this work may be developed as a detection tool for use along with the standard treatment of Malayan krait envenoming.

## 1. Introduction

In 2017, snakebite envenoming was once again recognized as a neglected tropical disease by the World Health Organization due to the lack of epidemiological surveys and sufficient prevention techniques, including clinical training and affordable antivenom/medications [1]. Envenoming following a venomous snakebite has become an increasing economic burden on the public health system in many tropical countries, especially in Sub-Saharan Africa, South Asia, Southeast Asia and South America [2,3,4].

In Thailand, there are three different species of krait. They are *Bangarus fasciatus* (banded krait), *Bungarus flaviceps* (red-headed krait) and *Bungarus candidus* (Malayan krait) [5]. Of these, *B. candidus* (Malayan krait) is classified as a category 1 venomous snake due to its widespread distribution and incidences of envenomings, which result in high levels of death and disability [6,7,8]. Progressive neuromuscular paralysis leading to respiratory failure is likely to be the most significant outcome observed following Malayan krait envenoming [9,10]. The mechanism behind this neuromuscular blockage is attributed to the inhibitory actions of presynaptic phospholipase A_2_ (PLA_2_)/beta (β) neurotoxins, postsynaptic/alpha (α) neurotoxins and three-finger toxins (3FTx) in krait venoms [11,12,13]. Moreover, abnormal blood pressure, tachycardia, elevated creatine kinase and electrolyze imbalance have also been reported in laboratory and clinical studies [14,15,16,17]. These life-threatening outcomes can be prevented and relieved by intubation and/or the early administration of specific antivenoms.

The Queen Saovabha Memorial Institute (QSMI) of the Thai Red Cross Society is the only snake antivenom manufacturer in Thailand. The antivenom is developed from the IgG of venom-immunized horses. The QSMI produces mono-specific snake antivenoms and neuro-polyvalent antivenom for Malayan krait envenoming. Clinically, antivenom is administered for neurotoxic snake envenoming as soon as one or two systemic neurotoxic symptoms, e.g., ptosis, external ophthalmoplegia or other skeletal muscle paralysis symptoms, are detected [18]. More antivenom than the standard recommendation (>50–100 mL) is necessary to treat krait envenoming due to presynaptic neuromuscular inhibition [17,19]. However, this antivenom can potentially cause severe allergic reactions or serum sickness. Techniques to measure the levels of snake venom in the blood of envenomed patients would help clinicians determine the appropriate amount of antivenom to administer for more effective therapeutic outcomes and fewer adverse reactions.

Previously, methods to detect and measure snake venom in envenomed victims involved several immunological assays, e.g., the enzyme-linked immunoassay (ELISA), the optical immunoassay and the avidin–biotin optical immunoassay [20,21,22,23]. However, the nonspecific reactivity of antibodies may alter the detection accuracy [24]. This might result from the longer antigen–antibody incubation time, which affects the sensitivity of the test. Moreover, low sensitivity and frequent false-positive results are significant problems in venom enzyme immunoassays [25]. In addition, a complex target SELEX-based identification of DNA aptamers was recently developed for use in the detection of *B. caeruleus* (common krait) envenoming [26].

Currently, electrochemical biosensors appear to be an essential tool for many biomedical diagnoses [27]. Electrochemical impedance spectroscopy (EIS) is a sensitive technique used to detect or measure biomolecular interactions on the surface of an electrode. Impedance changes are activated by incremental depositions on the surface of active electrodes, such as bioreceptors and target–bioreceptor complexes [28,29]. EIS is a promising method for quantifying molecules based on antibody reactions as recognition molecules on electrodes or cell impedimetric immunosensors, including bacteria, viruses, parasites and inflammatory markers [28,29,30,31]. This technique provides a powerful, informative and rapid electrochemical response; the target is not destroyed; the methods are easy to perform; and the process is inexpensive. Therefore, EIS has widespread applications [32]. If used clinically, the electrochemical biosensor technique would deliver more rapid results and data interpretation than conventional ELISA.

In this study, we applied an electrochemical technique using screen-printed gold electrodes (SPGEs) to measure *B. candidus* venom in experimentally envenomed rats. The knowledge obtained from this study can be developed for clinical applications in order to measure venom concentrations after snakebite envenoming.

## 2. Results

### 2.1. Examination of B. candidus Antivenom Specificity

#### 2.1.1. Dot Blot Hybridization and Indirect ELISA for *B. candidus* Venom Detection

Dot blotting and indirect ELISAs were performed to investigate the recognition of snake antivenoms containing venom-specific IgG and *B. candidus* venom.

**Figure 1:**
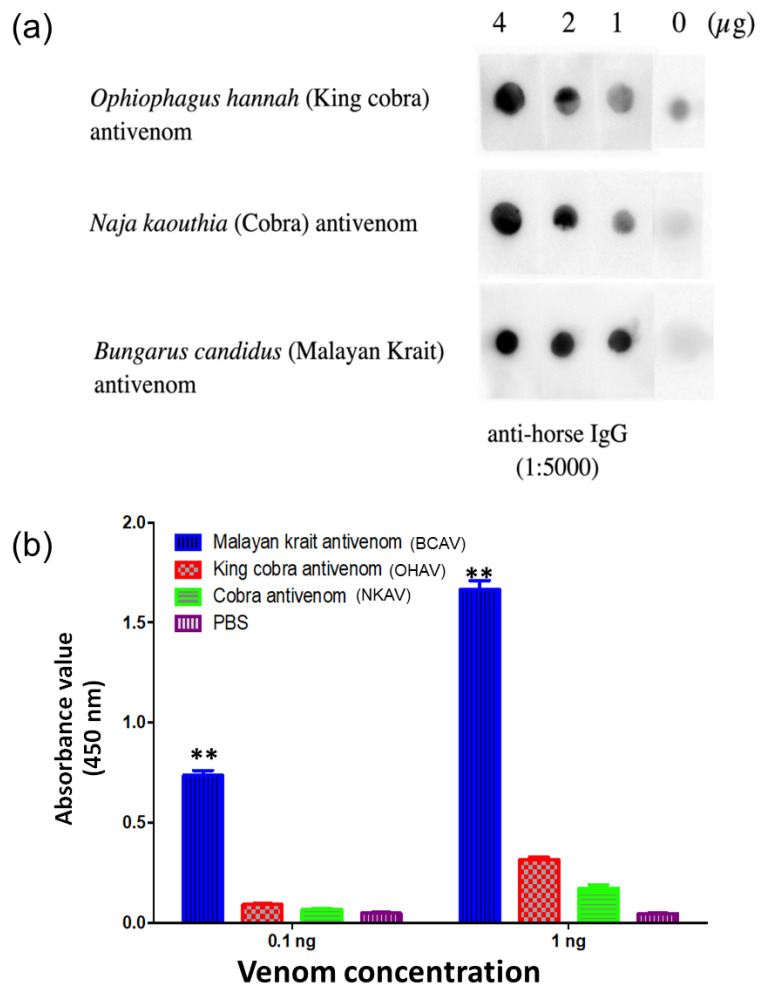
The binding capability of Malayan krait (*B. candidus*) venom to different antivenoms. (**a**) Dot blot hybridization and (**b**) indirect ELISA. ** indicates significantly different from NKAV, OHAV and phosphate-buffered saline (PBS) (*p*-value < 0.05, one-way ANOVA).

In dot blots, *B. candidus* venom (1, 2, 4 µg) was spotted onto nitrocellulose membranes and probed separately with different antivenoms (i.e., *B. candidus* antivenom, BCAV; *Ophiophagus hannah* antivenom, OHAV; and *Naja kaouthia* antivenom, NKAV; Figure 1a). The data showed different binding capacities between the three snake antivenoms and the *B. candidus* venom (Figure 1a and Figure S1). However, the *B. candidus* venom was detected by all the antivenoms. The highest density was detected in the chemiluminescence intensity between the *B. candidus* venom (1, 2 and 4 µg: Figure 1a) and the BCAV.

In an indirect ELISA, the *B. candidus* venom and BCAV exhibited the highest chemiluminescence compared with the OHAV and NKAV (Figure 1b). The A_450_ signal of the BCAV was also highest among all the monovalent antivenoms and showed a high sensitivity to the *B. candidus* venom at 0.1 and 1 ng.

#### 2.1.2. Sodium Dodecyl Sulfate–Polyacrylamide Gel Electrophoresis (SDS–PAGE) and Western Blotting

**Figure 2:**
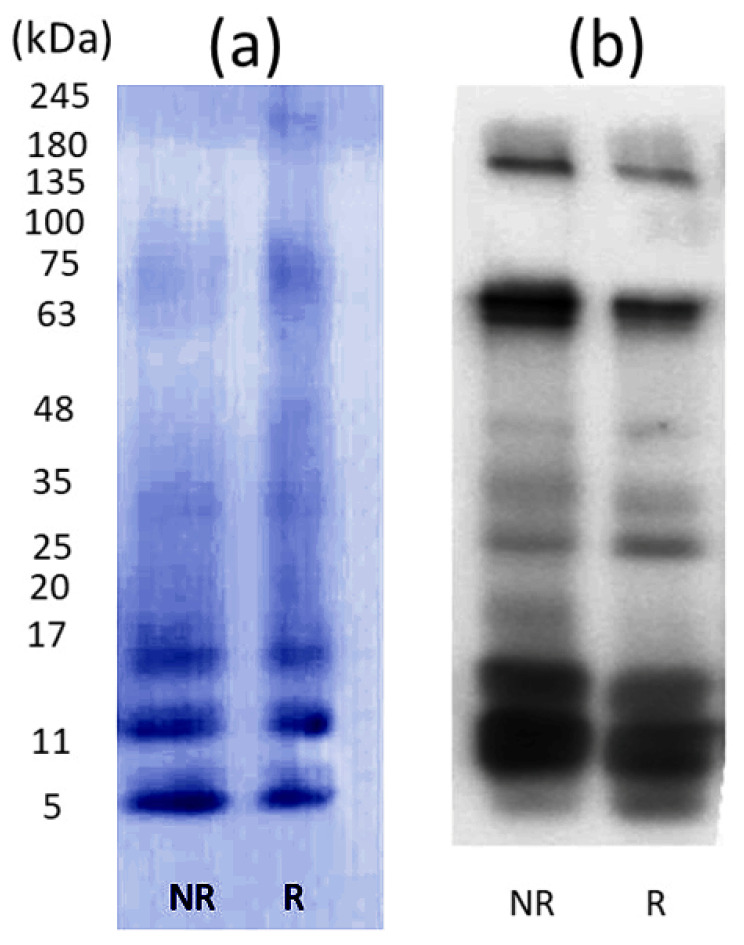
(**a**) SDS–PAGE and (**b**) Western immunoblot analysis of *B. candidus* venom on a 12% separating gel with a 5% stacking gel. Venoms were treated in nonreducing (NR) or reducing buffer (R) prior to loading and electrophoresis and stained with X-press Blue. Western immunoblotting of reduced *B. candidus* venom incubated with monovalent BCAV.

The SDS–PAGE analysis of the *B. candidus* venom indicated that the venom possessed thick and high-intensity bands in the MW range below 17 kDa under reduced and nonreduced conditions (Figure 2a). A low-intensity protein was observed within the range of 20–35 kDa in the reduced and nonreduced venoms with no remarkable differences (Figure 2a). A Western blot analysis (Figure 2b and Figure S2) indicated that most proteins in the venom were detected by the monovalent BCAV.

### 2.2. EIS Measurement for B. candidus Venom Detection under SPGE Biosensor

**Figure 3:**
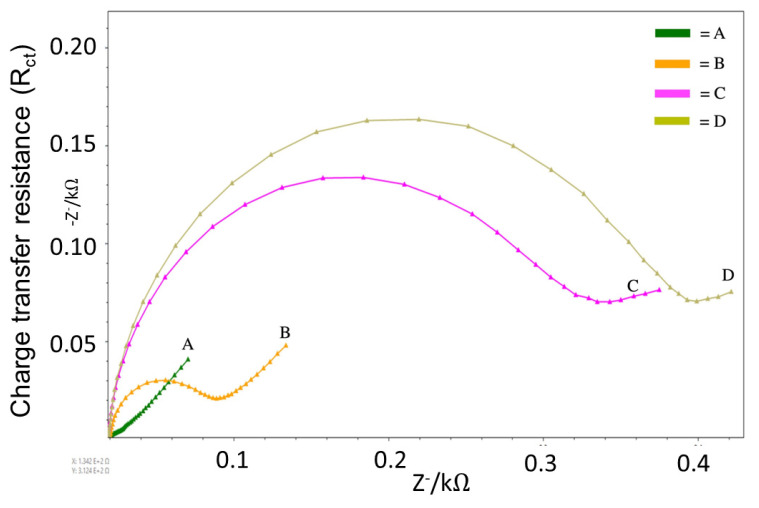
The EIS measurements show the changes in charge transfer resistance (R_ct_) in different modification steps of the SPGE biosensor, including (A) a bare electrode, (B) the BCAV (IgG)-coated electrode, (C) the BCAV (IgG)-coated electrode with blocking activity by mercaptohexanol and (D) following incubation with BC venom (0.1 mg/mL). Experiments were carried out in triplicate.

**Figure 4:**
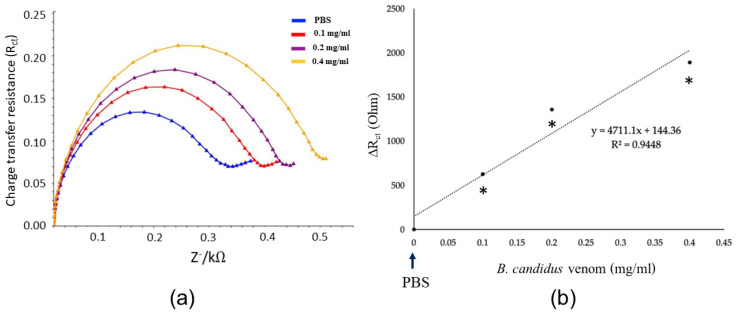
EIS measurement for *B. candidus* venom with the SPGE biosensor. (**a**) EIS measurements showed the Nyquist spectrum of the signal detected using the biosensor with different concentrations of *B. candidus* venom (0.1–0.4 mg/mL) and PBS (negative control). (**b**) Linear correlation between relative resistance and *B. candidus* venom concentration within the range of 0.1 to 0.6 mg/mL (PBS was used as a negative control). EIS was conducted in 5 mM of [Fe(CN_6_)]^3−/4−^ within the frequency range of 5.0 to 50,000 Hz. * indicates significantly different from the negative control (PBS, *n* = 3; *p*-value < 0.05, one-way ANOVA).

An increase in the charge transfer resistance (R_ct_) from the EIS measurement under the SPGE biosensor was observed following BCAV (0.1 mg/mL) incubation (immobilization; Figure 3). A significant increase in R_ct_ was observed following incubation with the *B. candidus* venom (0.2–0.4 mg/mL: Figure 4a). The EIS measurement data for each venom concentration were detected in triplicate. The correlation between R_ct_ and the *B. candidus* venom concentration was linearly related (Figure 4b, *n* = 3).

### 2.3. SWV Analysis of B. candidus Venom Concentration in Plasma from Non-Envenomed Rats

**Figure 5:**
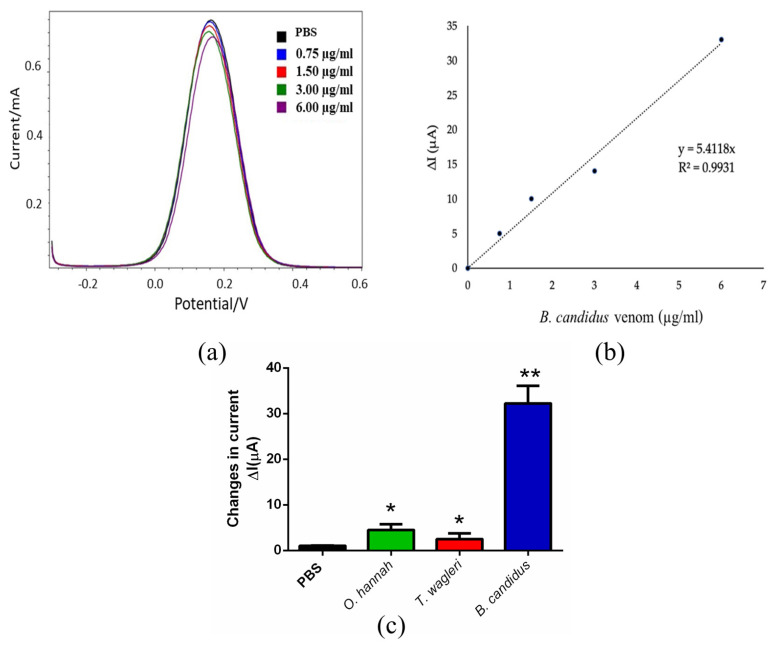
The SWV measurements of *B. candidus* venom. The different SWV currents (∆I) of the *B. candidus* venom in rat serum at various concentrations were detected (**a**) and plotted for the standard curve ((**b**); *n* = 3). (**c**) The different SWV currents (∆I) of the *B. candidus* venom (6 µg/mL) in rat plasma compared with the *O. hannah* and *T. wagleri* venoms (6 µg/mL) in serum. * indicates significantly different from PBS. ** indicates significantly different from the *O. hannah* and *T. wagleri* venoms, including PBS (*p*-value < 0.05, one-way ANOVA; *n* = 3).

To determine the ability of the biosensor to detect *B. candidus* venom in plasma, *B. candidus* venom (between 0.75 μg/mL and 6 μg/mL) was added to plasma from non-envenomed rats (Figure 5a). The amount of venom in the plasma was displayed as a change in the current (Figure 5a) under the SWV measurements. The increase in venom concentration was shown as a lower current peak compared to PBS. The *B. candidus* venom concentration in the plasma was calculated by using an equation of a standard curve that was plotted as the peak current change (μA) versus the venom concentration (μg/mL) (Figure 5b) (NB). The equation from the standard curve was also used for the *B. candidus* venom concentration in the envenomed rats (Section 2.4). The *B. candidus* venom (6 µg/mL) in the plasma was detected as a significant change in the SWV current, while the *O. hannah* and *T. wagleri* (*Tropidolaemus wagleri*) venoms (6 µg/mL) did not cause significant changes in the current, as determined by the SWV measurements (Figure 5c; *n* = 3).

### 2.4. SWV Analysis of B. candidus Venom Concentration in Plasma from Envenomed Rats

In the preliminary experiments, the *B. candidus* venom was administered to the rats at doses of 50, 100 and 200 µg/kg (i.v.; *n* = 3). The venom doses higher than 100 µg/kg resulted in the death of the rats within 2 to 3 h. Therefore, a dose of 100 µg/kg was chosen for further studies as it provided an optimal time frame for the study.

**Figure 6:**
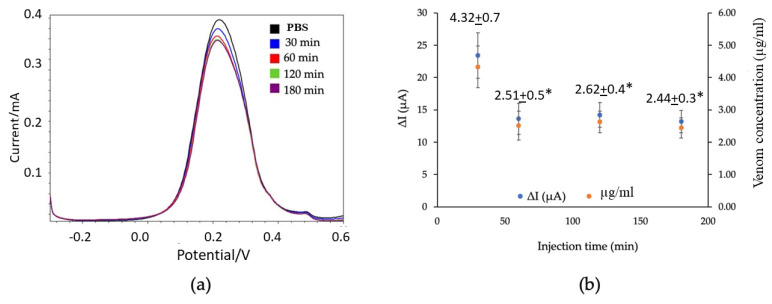
(**a**) SWV current signal from a rat serum sample measured at different time points, as indicated, after *B. candidus* venom administration, and (**b**) average SWV current change (∆I) versus the concentration of *B. candidus* venom in the rat plasma (*n* = 5) (**b**). * indicates significantly different from plasma collected 30 min after the administration of the *B. candidus* venom (*p*-value < 0.05, one-way ANOVA).

In the anesthetized rats, plasma was collected 30, 60, 120 and 180 min after the administration of the *B. candidus* venom (100 µg/kg, i.v., Figure S3). The electrochemical measurements showed a decrease in the SWV current relative to the time of sample collection (Figure 6a). The lowest SWV current was detected in the plasma collected 30 min after the *B. candidus* venom administration. In comparison, the rat plasma collected 60, 120 and 180 min after the *B. candidus* venom administration did not show marked differences in the SWV current (Figure S3). Based on calculations using the data from the standard curve, the maximum venom concentration was detected in a plasma sample collected after 30 min (4.3 ± 0.7 µg/mL; *n* = 5; Figure 6b). The lowest venom concentration was found in a plasma sample collected after 60 min (2.4 ± 0.3 µg/mL; *n* = 5; Figure 6b).

## 3. Discussion

Monovalent BCAV has been reported to minimize hospitalization time for patients envenomed by *B. candidus* in Thailand [33]. In addition, while *B. fasciatus* monovalent antivenom (BFAV) has been shown to neutralize the effects of three species of kraits in Thailand [34], neither BFAV nor BCAV were able to cross-neutralize the in vitro skeletal muscle effects of venoms from other *Bungarus* species [35].

Several snake venom enzyme immunoassays (EIAs) have been previously investigated to identify species and venom levels [20,21]. However, poor sensitivity and potential false-positive results are significant drawbacks when detecting and measuring venom concentrations in blood samples from envenomed victims. Moreover, the long antigen–antibody incubation time of conventional ELISAs is likely to be a limitation when a measurement of circulating snake venom levels is urgently needed. In this study, we applied screen-printed gold electrodes (SPGEs) as electrochemical biosensors for measuring *B. candidus* venom concentrations in plasma from experimentally envenomed rats. This device can be further developed as an effective tool to help clinicians promptly monitor snake venom in envenomed patients.

As the SPGEs must be incubated with BCAV for the characterization of electrochemical biosensors, we confirmed the specificity of the BCAV using dot blot hybridization, indirect ELISA and Western immunoblotting. Overall, the BCAV exhibited high specificity and bound almost every protein band of *B. candidus* venom in Western immunoblotting. The *B. candidus* venom–BCAV specificity was also confirmed by dot blot hybridization and an indirect ELISA. However, low binding activity between the *B. candidus* venom and the OHAV or NKAV was observed, suggesting some cross-reactivity; this might occur because the composition of the *B. candidus* venom may exhibit some similarities in venom compositions, e.g., three-finger toxins, Kunitz-type serine protease inhibitors, phospholipase A_2_ or phosphodiesterase [11,36,37]. The cross-reactivity of snake antivenom and other snake venoms in the enzyme immunoassay may cause difficulty in distinguishing different snake venoms, especially at low concentrations [25]. The purification of specific antibodies to major toxic components in *B. candidus* venom might be a solution for this cross-reactivity problem.

In the present study, electrochemical impedance spectroscopy (EIS) was used to characterize the reactivity between *B. candidus* venom and BCAV. EIS is a powerful technique used to analyze interfacial properties related to biorecognition events that occur at the electrode surface, such as antibody–antigen recognition, substrate–enzyme interactions, or whole-cell capture [32]. Hence, EIS could be applied to several biomedical diagnoses, especially in the areas of parasitology, virology and microbiology. To the best of our knowledge, in this study, we used EIS for the first time to measure the concentration of *B. candidus* venom. We initially evaluated the ability of BCAV-coated SPGEs to detect *B. candidus* venom by incubating them with a *B. candidus* venom solution (0.1–0.4 mg/mL). Their venom-detecting ability was presented as an increase in charge transfer resistance (R_ct_). The increase in R_ct_ after IgG (BCAV) immobilization indicates that the antibody was successfully immobilized on the electrode surface, whereas a higher increase in R_ct_ was found following venom incubation (Figure 3). This effect occurs due to the reduction in the electron transfer rate caused by the activation of the redox process and indicates that the BCAV interrupted the access of redox species to the transducer surface and blocked electron transfer [38]. This result suggests that the binding activity between the antibody and the *B. candidus* venom complex hinders the electron transfer process.

To measure *B. candidus* venom in rat plasma, square wave voltammetry (SWV) was chosen along with EIS measurements. Initially, varying concentrations of *B. candidus* venom (0.75–6.0 µg/mL) were applied to obtain the standard curve of the snake venom level in non-envenomed rat plasma. Following the intravenous administration of the *B. candidus* venom (100 µg/kg, equivalent to 30 µg in a rat weighing 300 g), plasma samples were collected after 30, 60, 120 and 180 min. The decreasing SWV current signal was related to the increasing venom concentration. The cross-reactivity between the *B. candidus* venom biosensor and the other snake (i.e., *O. hannah* and *T. wagleri*) venoms was not observed, as a significant change in the SWV current failed to be detected following the test using unenvenomed rat plasma mixed with the *O. hannah* and *T. wagleri* venoms. This suggests the specificity of the SPGE biosensor for *B. candidus* venom. Minimal changes in the SWV currents after the application of the plasma with the *O. hannah* and *T. wagleri* venoms were detected, suggesting that these snake venoms share the same enzymatic composition [12]. The BCAV–venom complex on the surface of the SPGEs was blocked and hindered the electron transfer reaction, resulting in a reduction in the SWV current. The SWV current (∆I) was calculated as ∆I = I_0_ − I_1_, where I_0_ and I_1_ refer to the current before and after incubation with the venom, respectively. Our data showed that the lowest SWV current was detected in plasma that was collected after 30 min, suggesting the highest amount of *B. candidus* venom in the blood circulation of experimentally envenomed rats. A fluctuation in the plasma venom concentration was observed (especially after envenoming for 60 min), which could result from snake venom pharmacokinetics and the route of administration [39]. A plateau in the serum venom concentration starting from 120 min following envenoming was also demonstrated [39]. Other mechanisms may be involved in addition to absorption and distribution, such as binding to some structures in the central compartment [39]. However, there were some limitations regarding using unconscious animals in this study, as the anesthesia may alter physiological processes, i.e., blood pressure or heart rate, following *B. candidus* venom administration [16]. Further investigations need to be performed in larger conscious animals, such as rabbits, and a more frequent sample collection for at least 24 h is required. Moreover, further clinical investigations are needed prior to applying the biosensor to envenomed victims, especially information regarding the limitations of the SPGE biosensor in detecting snake venom in patient plasma, which may have a variation in venom concentrations. In the current study, *B. candidus* venom (100 µg/kg, IV) was only applied to the experimental rats, which may not represent the actual amount of snake venom in humans. A clinical trial performed by using plasma from *B. candidus*-envenomed patients would confirm the reliability of this biosensor in clinical applications.

## 4. Conclusions

In the present study, a biosensor using SPGEs was developed for the detection of *B. candidus* venom with electrochemical characterization by either EIS or SWV, and this sensor can detect *B. candidus* venom in the serum of experimentally envenomed animals. The SPEG biosensor is a promising tool for clinical diagnosis and forensic investigation. However, there might be some aspects regarding antibody specificity and sensitivity that need to be further investigated. The accuracy and reliability of the biosensor for measuring snake venom concentrations in humans need to be further evaluated. This technique might be an effective approach to helping clinicians monitor and choose the appropriate snake venom treatment for Malayan krait-envenomed victims.

## 5. Materials and Methods

### 5.1. Venom Preparation and Antivenom

Freeze-dried Malayan krait (*B. candidus*) venom, monovalent *B. candidus* antivenom (BCAV; Lot No.: BC00115), *Ophiophagus hannah* antivenom (OHAV; Lot No. LH00120) and *Naja kaouthia* antivenom (NKAV; Lot No. NK00321) were obtained from Queen Saovabha Memorial Institute (QSMI) of the Thai Red Cross Society, Bangkok, Thailand. *Tropidolaemus wagleri* venom was a gift from Dr. Muhamad Rusdi Ahmad Rusmili (International Islamic University Malaysia). The venoms were milked from several snakes collected in Thailand and Malaysia by directly attaching a microhematocrit tube to each fang. Pooled venom was transferred to a 1.5 mL microcentrifuge tube, frozen at −20 °C and freeze-dried. The venom samples were then weighed, labeled and stored at −20 °C. When needed, the venom was weighed and dissolved in distilled water. The venom remained on ice during the experiments.

### 5.2. Animal Ethics and Care

Male Sprague–Dawley rats, purchased from Nomura-Siam International Co., Ltd., (Bangkok, Thailand), were housed in stainless steel containers with free access to food and drinking water. All animal experiments were approved by the Subcommittee for Multidisciplinary Laboratory and Animal Usage of Phramongkutklao College of Medicine and the Institutional Review Board, Royal Thai Army Department, Bangkok, Thailand (Documentary Proof of Ethical Clearance No. IRBRTA S077b/64_Xmp; date of approval; 23 September 2021) in accordance with the U.K. Animal (Scientific Procedure) Act, 1986, and the National Institutes of Health Guide for the Care and Use of Laboratory Animals (NIH Publications No. 8023, revised 1978).

### 5.3. Anesthetized Rat Preparation and Blood Sample Collection

This procedure was performed as previously described [16]. Briefly, male Sprague–Dawley rats weighing 300–350 g were anesthetized using Zoletil^®^ (20 mg/kg, i.p.) and Xylazine^®^ (5 mg/kg, i.p.). Additional anesthetic was administered as required. A midline incision was made in the cervical region, and a cannula was inserted into the right jugular vein for venom administration. A carotid artery was inserted for the monitoring of blood pressure and sample collection, and a tracheal cannula was inserted for artificial respiration, if required. Blood pressure was recorded using a pressure transducer filled with heparinized saline (25 U/mL) and monitored on a MacLab system (ADInstruments). If needed, normal saline (100 µL, 0.9% NaCl) was administered (i.v.) to maintain the blood volume. The body temperature of the rats was maintained by a heat lamp. At various time points (i.e., 30, 60, 120 and 180 min post-injection of venom or saline), 0.5 mL of blood was collected from the carotid artery in Eppendorf tubes. The samples were centrifuged at 5500 rpm for 10 min, the supernatant was stored at −20 °C for no longer than 12 h, and the levels of *B. candidus* venom were determined. At the conclusion of the experiment, the animals were euthanized by an overdose of anesthetic agents (i.v.).

### 5.4. Dot Blot Hybridization and Indirect ELISA for Snake Venom Antigen Detection

*B. candidus* venom was serially diluted by 2-fold (1, 2, and 4 µg) with PBS and spotted onto nitrocellulose membranes separately. Excess snake venom was removed by extensive washing and blocked with 5% skim milk for 1 h at 4 °C. After washing, 10 µg/mL of each snake monovalent antivenom (i.e., *B. candidus* antivenom; Lot No. BC00115: BCAV, *Ophiophagus hannah* antivenom; Lot No. LH00120: OHAV, *Naja kaouthia* antivenom; Lot No. NK00321: NKAV, Queen Saovabha Memorial Institute of the Thai Red Cross) was incubated with antigen-immobilized nitrocellulose membrane at 4 °C for 1 h. The snake venom and snake antivenom cross-reactivity was visualized with HRP-conjugated goat anti-horse IgG (1:5000, Thermo Fisher Scientific, Waltham, MA, USA). Finally, the membrane was detected with a LumiFlash™ chemiluminescent substrate (Enegenesis Biomedical, Taipei, Taiwan). In addition, an indirect ELISA was performed to determine antigen recognition. Briefly, *B. candidus* venom (0.1, 1 ng) was coated on a 96-well plate as the target antigen. After washing and blocking, 10 µg/mL of BCAV, NKAV and OHAV were added to the immobilized antigen wells separately. Then, HRP-conjugated goat anti-horse IgG (1:10,000) was added and incubated for 1 h. After washing, the colorimetric reaction was developed with a TMB substrate, followed by 1 N of HCl to stop the reaction. The reaction was quantified by measuring the absorbance at 450 nm (A_450_).

### 5.5. Sodium Dodecyl Sulfate–Polyacrylamide Gel Electrophoresis (SDS–PAGE)

The *B. candidus* venom (10 μg) in reducing and nonreducing sample buffers was resolved and electrophoresed at 90 V in a 12% separating gel with a 5% stacking gel [40]. Protein bands were visualized by staining with X-Press Blue Protein Stain (Himedia, Kennett Square, PA, USA), followed by destaining with distilled water. A TriColour Broad Protein Ladder (Biotechrabbit GmbH, Henigsdorf, Germany) was electrophoresed in the gel as a protein molecular weight marker. The gel was scanned using a Chemi Imager, Alliance Mini HD9 Auto (UVITEC, Cambridge, UK) [41].

### 5.6. Western Immunoblotting

*B. candidus* venom (10 µg) was resolved on a 12% SDS–PAGE gel and transferred onto a polyvinylidene difluoride membrane (Merck Millipore, Billerica, MA, USA) using wet electroblotting (Cleaver Scientific, Warwickshire, UK) at 300 mA for 45 min. The membrane was then blocked in 5% skim milk in TBST (20 mM Tris, 0.5 M NaCl, 0.5% Tween-20) to prevent nonspecific binding and incubated with a primary antibody (i.e., monovalent BCAV diluted 1:500-fold in TBST with 5% skim milk) overnight at 4 °C. The membrane was then washed with TBST buffer three times for 30 min. Immunoreactive bands were visualized using appropriate secondary antibodies (horseradish peroxidase-conjugated rabbit-anti-horse-IgG, Sigma, UK) and a western chemiluminescence ECL detection reagent (Cyanagen Srl; Bologna, Italy). The membrane was scanned using a Chemi Imager, Alliance Mini HD9 Auto (UVITEC, Cambridge, UK).

### 5.7. Invention of Gold Electrode Biosensor for Measuring B. candidus Venom Concentration

**Figure 7:**
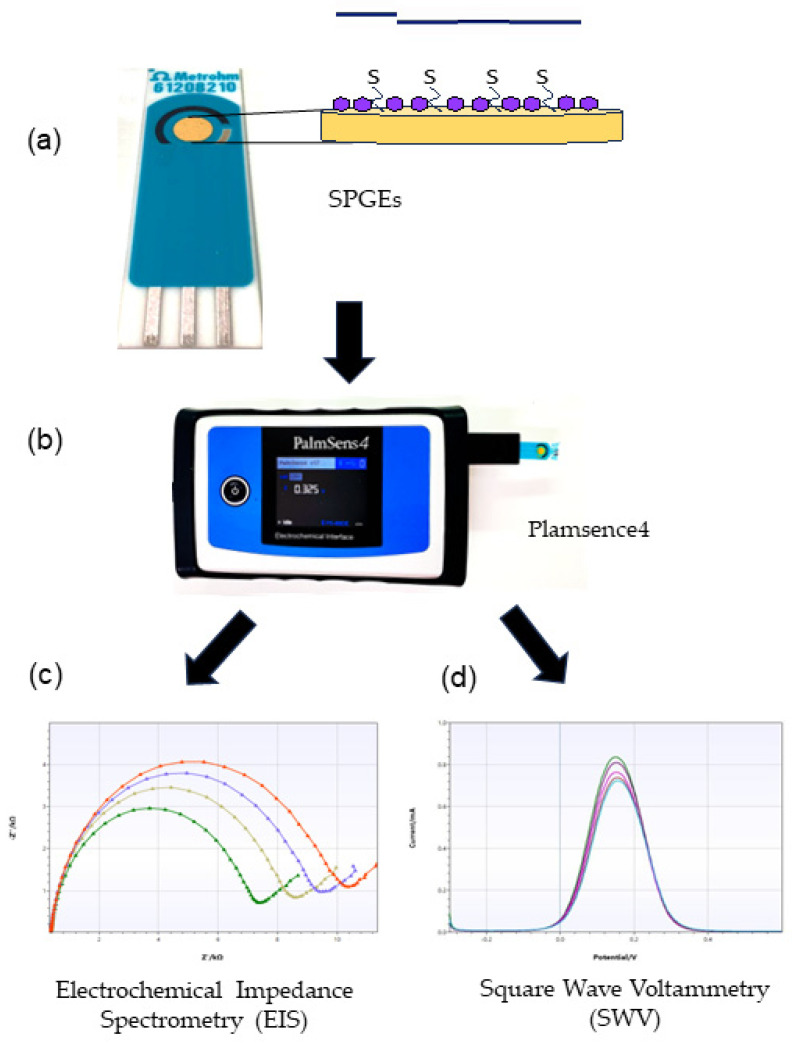
Diagram of the electrochemical biosensor: (**a**) SPGE biosensor; (**b**) Plasmence4; (**c**) EIS measurement; and (**d**) SWV measurement.

BCAV (5 µL: 50 µg/mL) was immobilized onto active screen-printed gold electrode (SPGE: Metrohm, 6.1208.210, LB Barendrecht, The Netherlands; Figure 7a) surfaces and incubated at 4 °C overnight. The electrodes were washed thoroughly with phosphate-buffered saline (PBS) to remove excess BCAV and blocked with mercaptohexanol (MCH: 5 μL of 0.5 mM) for 10 min at room temperature to reduce nonspecific binding. Then, the SPGEs were washed with distilled water and applied as a biosensor for *B. candidus* venom detection.

### 5.8. Electrochemical Analysis

Electrochemical characterization was carried out using a PalmSens4 potentiostat (PalmSens BV, GA Houten, The Netherlands; Figure 7b) and PS Trace 5.8 software. The SPGE biosensor was washed with PBS before the measurements and activated with cyclic voltammetry (CV). CV was applied with a potential of −0.3 V to 0.6 V at a scan rate of 50 mVs^−1^ using a redox indicator. To determine the snake-venom-detecting ability of the SPGEs, different concentrations of venom or collected rat plasma were incubated with the SPGE biosensor for 10 min and then rinsed with PBS. Afterwards, 100 µL of 5 mM [Fe(CN)_6_]^4−/3−^ was added, and all the electrode surfaces were immersed. Biosensor characterization was performed by electrochemical impedance spectrometry (EIS, Figure 7c) and square wave voltammetry (SWV, Figure 7d). The EIS measurements were performed by applying a current range of 100 nA–100 mA with the scan type fixed at E_dc_ = 0.00 V and E_ac_ = 0.006, a frequency of 50,000–5.0 Hz and t Max. The OCP was 1.0 s, and the stability criterion was 0.0 mV/s. The SWV measurements were performed by applying a potential range of −0.3 to 0.6 V with a step potential of 3 mV, an amplitude of 80 mV and a frequency of 8 Hz.

### 5.9. Characterization of the Electrochemical Biosensor by EIS

The electrochemical sensor modified on the gold electrode surface was characterized by EIS using 5 mM of [Fe(CN)_6_]^4−/3−^. The EIS value exhibited changes the charge transfer resistance (R_ct_) in each modification step (Figure 3). The equivalent electrical circuit fitting in this experiment was a mixed kinetic and diffusion control (Randles cell) circuit [42], consisting of a series of active electrolyte resistors (R_s_) and a parallel combination of double layer capacitors (C_dl_) connected to the charge transfer resistance (R_ct_) and the Warburg impedance (W) of a faradaic reaction (Figure S4). The Warburg coefficient, σ, is 1 kΩ·s^−1/2^ in this case, and the other values are R_s_ = 1 kΩ, R_ct_ = 1 kΩ, and C_dl_ = 10 nF.

### 5.10. Data Analysis and Statistics

The statistical analysis of the data was carried out using a one-way analysis of variance (ANOVA) followed by a Bonferroni multiple comparison test, where a *p*-value < 0.05 was considered statistically significant (Prism 6.0; GraphPad Software, La Jolla, CA, USA). The data are expressed as the mean ± SEM.

## Data Availability

The data presented in this study are available on request from the corresponding author. The data are not publicly available due to privacy policy.

## Supplementary Materials

The following supporting information can be downloaded at https://www.mdpi.com/article/10.3390/toxins16010056/s1: Figure S1: Original dot blot; Figure S2: Original SDS = PAGE and Western immune blotting; Figure S3: The SWV measurement curve of *B. candidus* venom measured after being injected into rats at 30 (a), 60 (b), 120 (c) and 180 (d) min on individual biosensors; Figure S4: Warburg diffusion element.
