# Supplementary material for: Development of a Biosensor to Detect Venom of Malayan Krait (Bungarus candidus)
Source: Toxins (Basel). 2024 Jan 19;16(1):56. doi: 10.3390/toxins16010056 (PMC10820552; doi:10.3390/toxins16010056)
Supplement: Supplementary file 1 [file toxins-16-00056-s001.zip › toxins-2753640-supplementary.pdf]

Supplementary Materials: Development of a Biosensor to Detect Venom of Malayan Krait (*Bungarus candidus*)

Kiattawee Choowongkomon, Janeyuth Chaisakul, Supaphorn Seetaha, Taksa Vasaruchapong, Wayne C. Hodgson, Natchaya Rasri, Katechawin Chaeksin, Sattawat Boonchaleaw and Nattapon Sookprasert

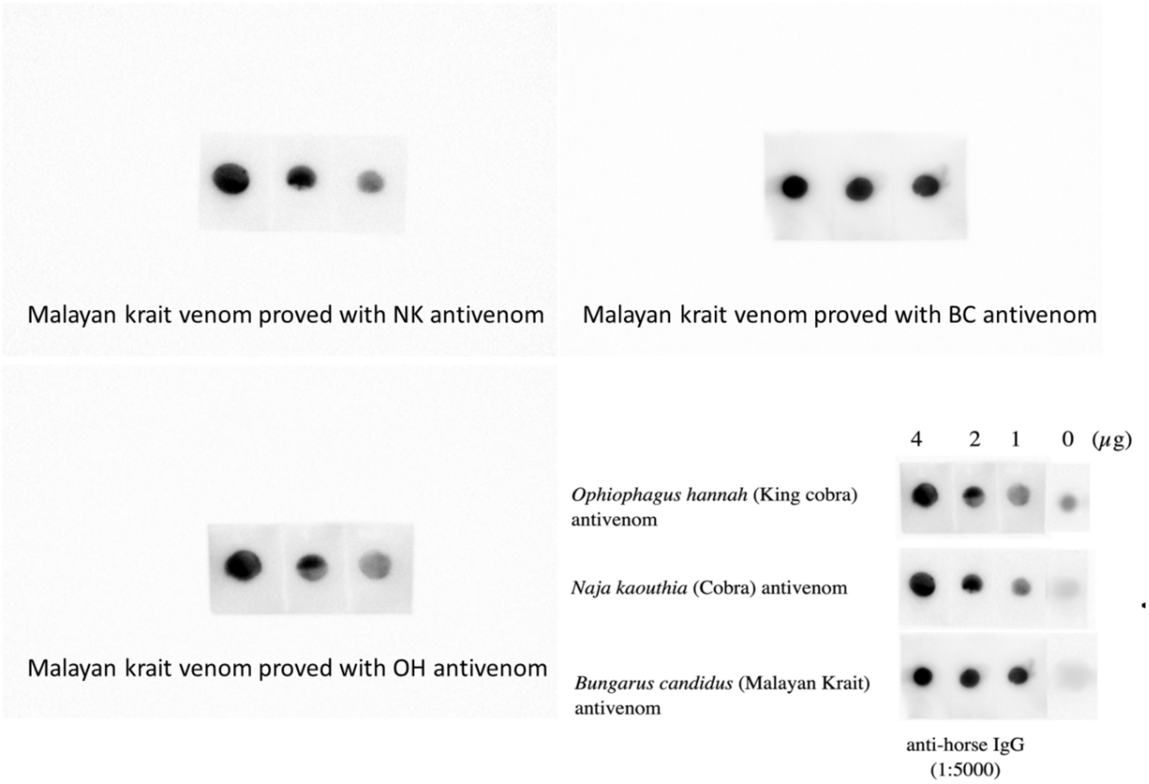

Figure S1: Original dot blot.

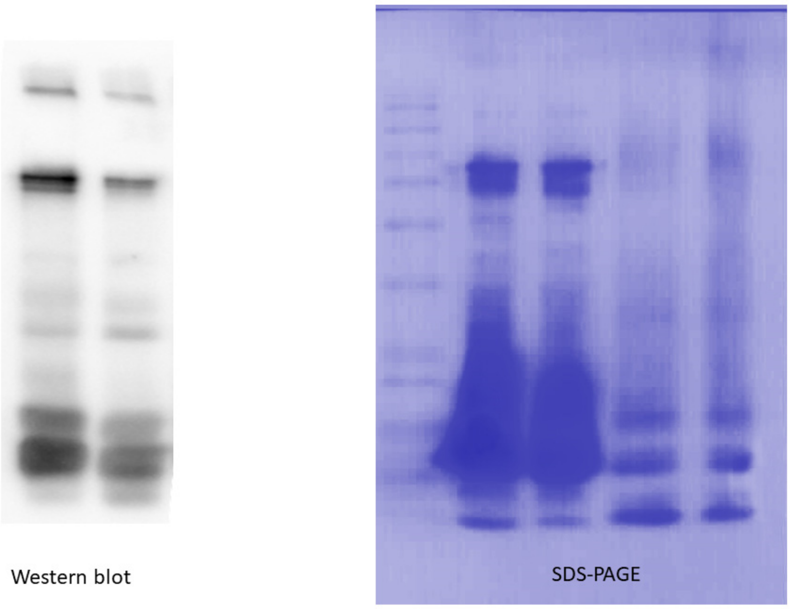

Figure S2: Original SDS=PAGE and Western immune blotting.

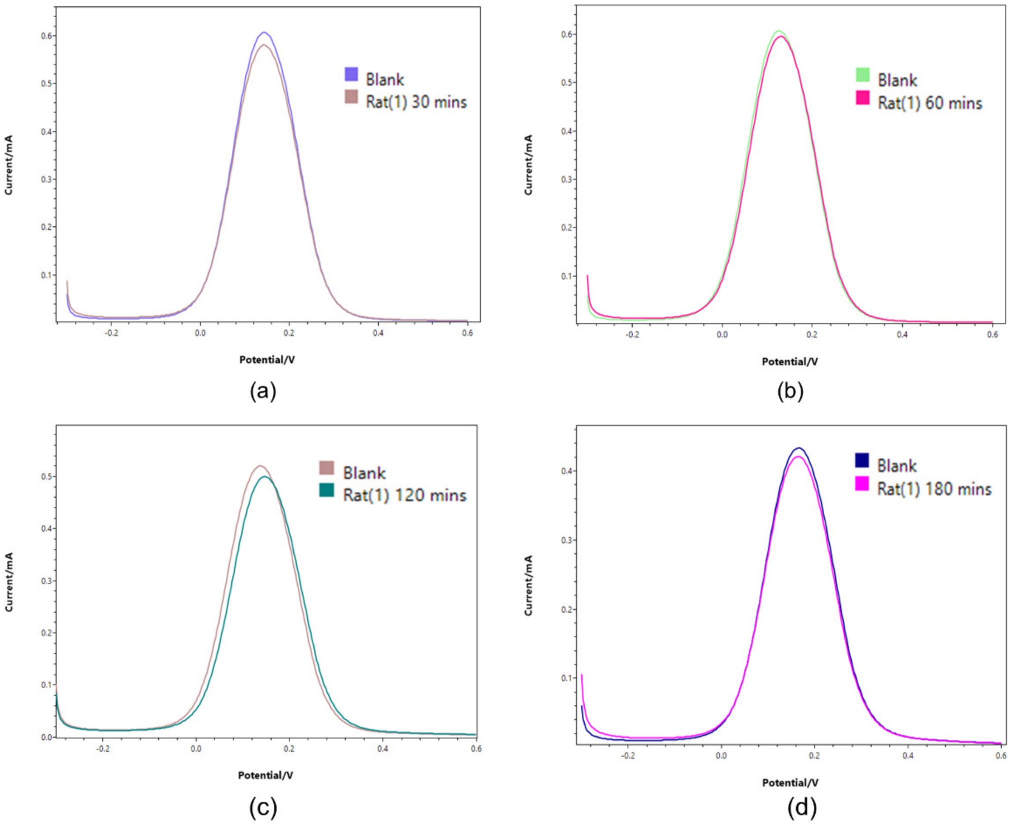

Figure S3: The SWV measurements curve of *B. candidus* venom measured after injected into rat at 30 (a), 60 (b), 120 (c) and 180 (d) min on individual biosensor.

Warburg diffusion element

Circuit Fitting : -Z Imaginary / Z Real

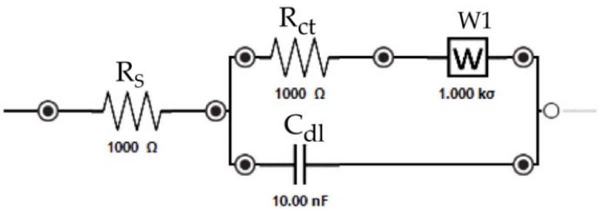

$$Z_{\mathrm{W}} = \frac{A_{\mathrm{W}}}{\sqrt{\omega}} + \frac{A_{\mathrm{W}}}{j\sqrt{\omega}}$$

Equivalent circuit : R([RW]C)

R = Resistor

W = Warburg element

C = Capacitor

- A_W is the Warburg coefficient (or Warburg constant)
- j is the imaginary unit
- ω is the angular frequency

Figure S4: Warburg diffusion element.
